# Knowledge and Preventive Practices About Osteoporosis Among Elementary School Teachers of Bandar-Abbas in 2020

**DOI:** 10.3389/fnut.2022.849639

**Published:** 2022-04-04

**Authors:** Ali Nikoobar, Ali-Asghar Kolahi

**Affiliations:** Social Determinants of Health Research Center, Shahid Beheshti University of Medical Sciences, Tehran, Iran

**Keywords:** awareness, behavior, diet, exercise, osteoporosis, school teachers (MeSH)

## Abstract

**Purpose:**

To assess knowledge and preventive practices about osteoporosis among elementary school teachers of Bandar-Abbas in 2020.

**Materials and Methods:**

In this cross-sectional study, the schools were selected using simple random sampling, and the teachers were invited to fill out an online questionnaire. The knowledge section of the questionnaire assessed general knowledge and knowledge regarding risk/protective factors, nutrition, and physical activity. The preventive practices section assessed dietary habits using a food frequency questionnaire, asking about 16 items in six groups, intakes of which were compared to the recommendations of the Iranian food pyramid. This section also assessed physical activity using the International Physical Activity Questionnaire.

**Results:**

Totally, 377 school teachers with a mean [standard deviation (SD)] age of 38 (6.7) participated in this study. The level of overall knowledge of 128 (33.9%) teachers was high, 222 (58.9%) moderate, and 27 (7.2%) low. The median [interquartile range (IQR)] intakes of fruits [2 (1–2)] and meats/eggs [1.7 (1.4–2.2)] were adequate, while those of dairy products [1.5 (0.9–2.3)], nuts/legumes [0.5 (0.2–1.1)], and vegetables [0.3 (0.1–1)] were inadequate. The median (IQR) intakes of tea/coffee [1 (0.6–2)] and cola [0.1 (0–0.3)] were considered limited. The physical activity level of 121 (32.1%) teachers was high, 124 (32.9%) moderate, and 135 (35%) low.

**Conclusion:**

Knowledge of the teachers about osteoporosis was moderate, and their preventive practices were somewhat adequate concerning dietary habits and moderate concerning physical activity.

## Introduction

Osteoporosis is a growing health problem worldwide, causing over 8.9 million fragility fractures annually (1, 2). This estimated number has been, and is, on the rise due to the increase in the prevalence of osteoporosis, which is mainly due to the worldwide aging of the population and changing lifestyle habits (2). The global incidence of hip fracture, the most severe osteoporotic fracture, is projected to increase by 232%, from 2.7 million cases in 2010 to 6.26 million in 2050 (2–4). In Iran, based on a meta-analysis in 2013, the prevalence of osteoporosis is 12, 3, and 19% among men and pre- and postmenopausal women, respectively (5). The annual incidence of hip fractures in Iran is 138.3 and 157.5 per 100,000 persons in men and women, respectively, according to a meta-analysis in 2021 (6).

About 80% of these patients with osteoporosis are neither identified nor treated after the first fracture, leading to a second fracture in many of them and an excess cost (7, 8). Therefore, despite the various treatments available, prevention remains the most cost-effective tool in fighting osteoporosis (9, 10). The prevention of osteoporosis is mainly achieved through having a healthy diet and enough physical activity and avoiding its modifiable risk factors, including smoking, excessive alcohol intake, and frequent falls (11, 12). A healthy diet, including dairy products, fruits, vegetables, animal protein, etc., provides the bones with nutrients essential for their structure and function, such as calcium, vitamins, and protein. On the other hand, coffee, tea, and cola may have deleterious effects on bone mineral density at excessive intakes (11, 13, 14). Enough physical activity, especially weight-bearing, induces bone and muscle building and strength by stressing them; it also improves strength and balance to help prevent falls (11).

In this sense, knowledge about osteoporosis and these preventive practices are targeted to be promoted among many groups, such as employees, schools, students, and parents in the “Osteoporosis strategic plan for the Middle East and North Africa region” (15). School teachers, as school employees themselves, as important role models for students and as those in direct contact with parents, could play a significant role in implementing this plan (16, 17). Moreover, school teachers, except for physical education teachers, are at risk of low or moderate physical activity and poor diet. Since they are usually sitting or standing during the working time, and as they work hard, they cannot spend much time having a healthy diet and enough physical activity during their leisure time (18). By promoting these practices, school teachers, as adults, could maintain their bone mass and avoid premature bone loss (11). Nevertheless, before promotion, we need to have baseline information concerning knowledge and preventive practices about osteoporosis among this group to prioritize the programs, especially those concerning worksite wellness (15, 19). To our knowledge, few studies have assessed these targets among school teachers, reporting low knowledge, moderate knowledge, and poor practices among female teachers of Rafsanjan, Shahrekord, and Rasht, respectively (20–22). Therefore, the purpose of this study was to assess knowledge and preventive practices about osteoporosis among elementary school teachers of Bandar-Abbas in 2020.

## Materials and Methods

### Setting

This descriptive cross-sectional study was conducted from January to September 2020 among elementary school teachers of Bandar-Abbas, a port city positioned overlooking the entrance of the Persian Gulf in Hormozgan Province in southern Iran. The population of Bandar-Abbas is 680,366, according to the latest census in 2017 (23).

### Participants

**FIGURE 1:**
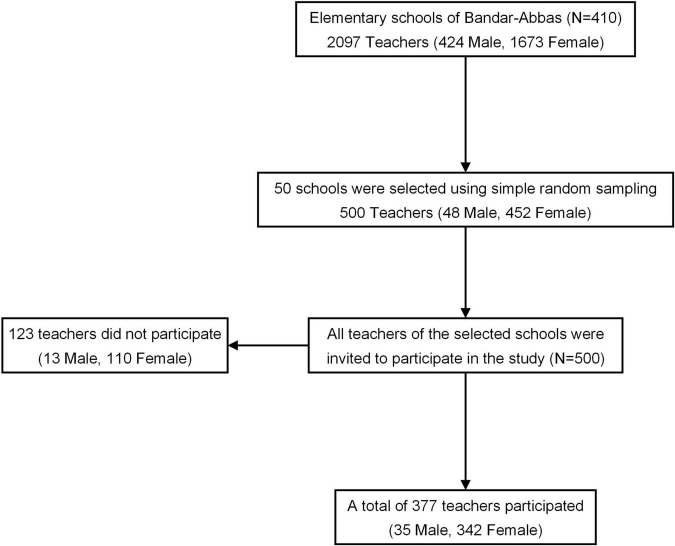
Flow chart of selecting the participants among elementary school teachers of Bandar-Abbas.

A total of 500 teachers out of the 2,097 elementary school teachers of Bandar-Abbas were invited to participate in the study, 377 of whom participated (Figure 1). First, 50 schools were selected out of the 410 elementary schools of Bandar-Abbas using simple random sampling. Then, all teachers of the selected schools were invited to fill out an online questionnaire by the principal, a vice-principal, or a teacher of each school. The invitation link was sent *via* WhatsApp messenger.

### Data Collection

Data were collected using an online self-administered questionnaire *via* Google Form, consisting of three sections: sociodemographic characteristics, knowledge, and preventive practices (Supplementary Material).

#### Sociodemographic Characteristics

The sociodemographic characteristics section included nine items: sex, age, years’ experience, height, weight, marital status, academic degree, university, and field of study.

#### Knowledge

The knowledge section included 19 items with three choices—“True,” “False,” and “Do not know” —in four subscales. The items were selected through a literature review, including the information provided by the International Osteoporosis Foundation (IOF) (24) and the items of the validated questionnaires. These questionnaires included the Osteoporosis Knowledge Assessment Tool (OKAT), the revised version of the Facts on Osteoporosis Quiz (FOOQ), and those of the studies conducted among Iranian female teachers and employees (20, 25–27).

The knowledge subscales included the following: (1) general knowledge, including five items about bone fractures, the name of osteoporosis, and signs and diagnosis of osteoporosis; (2) knowledge regarding risk/protective factors, including seven items about the effect of early menopause, smoking, age of 65 and above, sex, being thin, ethnicity, and high body mass index (BMI) on the risk of osteoporosis; (3) knowledge regarding nutrition, including four items about the role of calcium-rich foods, sun exposure, vitamin C-rich foods, and animal proteins in the prevention of osteoporosis, and (4) knowledge regarding physical activity, including three items about the role of enough physical activity, walking, and weight-bearing activities in the prevention of osteoporosis.

#### Preventive Practices

The preventive practices section consisted of two subsections: (1) Dietary habits subsection included a food frequency questionnaire (FFQ) asking about 16 food items: milk, yogurt and/or dough, cheese, ice cream, nuts, legumes, fruits, vegetables, red meat, chicken, seafood, egg, tea and/or coffee, and cola. These food items affect bone health according to the IOF, the National Osteoporosis Foundation (NOF), and “Krause’s Food & the Nutrition Care Process” textbook (11, 13, 14).

(2) Physical activity subsection included the long form of the International Physical Activity Questionnaire (IPAQ) in Persian (28), which was modified for school teachers of Bandar-Abbas. This questionnaire describes walking, moderate physical activity, and vigorous physical activity within four domains, including working time, transportation, yard and house, and leisure time in the last 7 days (29). As the job-related activities of the school teachers, except the physical education teachers, are done behind the desk, or in a standing position, question one was changed from “Do you currently have a job or do any unpaid work outside your home?” to “Are you a physical education teacher, or do you have a second job needing physical activity?” Other modifications included changes in some examples for different types of physical activity to match the everyday activities of school teachers of Bandar-Abbas.

#### Validity and Reliability

A panel of experts evaluated the content validity of the questionnaire. An item discrimination analysis was conducted for each scale to eliminate too tricky or easy items. Factor analysis was performed for factor structure. A separate test-retest over 2 weeks was held for the three sections of the questionnaire. The test-retest correlation for the knowledge section was 0.86; Kuder-Richardson-20 was used to prevent overestimating internal consistency; the coefficient was 0.85. The test-retest correlation for the FFQ was 0.80; the coefficient alpha was 0.89. The test-retest correlation for the modified long form of the IPAQ was 0.89; the coefficient alpha was 0.90. The pilot survey was conducted on ten male and ten female elementary school teachers recruited online *via* convenience sampling method.

### Data Analysis

Among the sociodemographic characteristics, the teachers’ BMIs were calculated using their self-reported heights and weights.

To score knowledge, first, correct answers were scored as “1,” while wrong answers and “Do not know” responses were scored as “0.” Then, the scores were summed in each knowledge subscale. Finally, scores of the four subscales were summed to calculate an overall knowledge score. To categorize knowledge scores, scores less than 50% of the total were categorized as “low,” scores of 50% and above, but less than 75% were categorized as “moderate,” and scores of 75% and above were categorized as “high.”

To analyze dietary habits, intakes of all food items were calculated in servings/day. Second, the food items were grouped into six food groups of the Iranian food pyramid, including dairy products, nuts and legumes, fruits, vegetables, meats and eggs, and “others.” Third, the intakes of dairy products, nuts and legumes, and meats and eggs were calculated by summing the intakes of the food items in each group. Finally, the intakes of the food groups were compared to the recommendations of the Iranian food pyramid for assessing their adequacy (30).

To analyze physical activity, minutes/week and metabolic equivalents (METs)-minutes/week values and physical activity levels were computed using the guidelines for the IPAQ data processing and analysis (29).

Statistical analyses were performed using IBM^®^ SPSS^®^ Statistics Version 26. Data were expressed as means [standard deviation (SD)] for age, years’ experience, and scores of knowledge, as medians [interquartile range (IQR)] for other continuous variables, and as frequencies [percentage (%)] for categorical variables. Data were also expressed as minimum and maximum values for age, years’ experience, and knowledge scores. Continuous and ordinal variables were assessed for normality using the Shapiro-Wilk test and Kolmogorov–Smirnov test. They were non-normally distributed and were also assessed for homogeneity of variance across the teachers’ groups by other variables using non-parametric Levene’s test. Continuous variables were compared between the teachers’ groups by sociodemographic characteristics and other variables using Mann-Whitney U and Kruskal-Wallis for two and k samples, respectively. However, variables violated for homogeneity of variance were compared using the independent sample *t*-test with bootstrapping and median test. Correlations between continuous and ordinal variables were assessed using Spearman’s rank correlation. Categorical and ordinal variables were compared using the Pearson chi-square test. A probability value of 0.05 or less was considered statistically significant.

## Results

Totally, 377 elementary school teachers participated in the study, around 90% of whom were female and/or married (Table 1). The mean (SD) age of the teachers was 38 (6.7), range = 20–63, and the mean (SD) years’ experience of them was 13.9 (8.1), range = 0–38.

**TABLE 1:** Sociodemographic characteristics of the teachers.

Variable	n (%)
**Sex**	
Female	342 (90.7)
Male	35 (9.3)
**BMI**	
Underweight	13 (3.4)
Normal-weight	170 (45.1)
Overweight	147 (39.0)
Obese	47 (12.5)
**Marital status**	
Never-married	28 (7.4)
Engaged	3 (0.8)
Married	334 (88.6)
Separated	9 (2.4)
Widowed	3 (0.8)
**Academic degree**	
Diploma	5 (1.3)
Associate’s	45 (11.9)
Bachelor’s	278 (73.7)
Master’s	48 (12.7)
**University**	
Islamic Azad	159 (42.2)
Farhangian	121 (32.1)
Payame Noor	41 (10.9)
Governmental universities	33 (8.8)
Applied Science and Technology	15 (4.0)
Non-governmental universities	3 (0.8)
Teachers’ college	5 (1.3)
**Field of study**	
Education	180 (47.7)
Humanities and social sciences	122 (32.4)
Natural sciences	23 (6.1)
Formal sciences	17 (4.5)
Physical education	16 (4.2)
Natural resources	8 (2.1)
Engineering sciences	6 (1.6)
Medical sciences	4 (1.1)

### Knowledge

The mean (SD) overall knowledge score of the teachers was 13.3 (2.5) out of 19, and the level of overall knowledge score of above 90% of the teachers was moderate or high (Table 2).

**TABLE 2:** Knowledge scores among the teachers.

Knowledge subscale	Mean (SD) score	Min.–Max. scores	Score level, n (%)		
			Low	Moderate	High
General knowledge (total = 5)	3.6 (1.1)	1–5	62 (16.5)	114 (30.2)	201 (53.3)
Knowledge regarding risk/protective factors (total = 7)	4.6 (1.3)	1–7	64 (17.0)	218 (57.8)	95 (25.2)
Knowledge regarding nutrition (total = 4)	2.9 (0.9)	1–4	20 (5.3)	92 (24.4)	265 (70.3)
Knowledge regarding physical activity (total = 3)	2.1 (0.9)	0–3	88 (23.3)	127 (33.7)	162 (43.0)
Overall knowledge (total = 19)	13.3 (2.5)	6–19	27 (7.2)	222 (58.9)	128 (33.9)

Regarding general knowledge, around 90% of the teachers or above knew about bone fractures and the association of osteoporosis with them (Table 3). However, only around half knew whether osteoporosis has any evident signs before the first fracture. Regarding risk/protective factors, above 80% of the teachers knew about the effect of early menopause, smoking, old ages, and/or sex on the risk of osteoporosis. Regarding nutrition, all of the teachers knew about the role of calcium-rich foods in preventing osteoporosis, while only half of them knew about the role of animal protein. Regarding physical activity, around 80% of the teachers knew about the role of enough physical activity and/or walking to prevent osteoporosis, while around half knew about the role of weight-bearing physical activities.

**TABLE 3:** Knowledge items and responses of the teachers to them.

Knowledge item by knowledge subscale	Correct answer (%)	Wrong answer (%)	“Do not know” (%)
**General knowledge**			
Osteoporosis increases the risk of bone fracture.	366 (97.1)	3 (0.8)	8 (2.1)
In a person with osteoporosis, a previous bone fracture increases the chance of another bone fracture.	340 (90.2)	16 (4.2)	21 (5.6)
Osteoporosis is also named “Rheumatism.”	260 (69.0)	57 (15.1)	60 (15.9)
Osteoporosis does not usually have a clear sign until the first bone fracture occurs.	191 (50.7)	130 (34.5)	56 (14.8)
A blood test must be used for the diagnosis of osteoporosis.	183 (48.5)	101 (26.8)	93 (24.7)
**Knowledge regarding risk/protective factors**			
The chance of developing osteoporosis is higher in women with early menopause.	338 (89.6)	7 (1.9)	32 (8.5)
Smoking increases the risk of osteoporosis.	332 (88.1)	17 (4.5)	28 (7.4)
The risk of osteoporosis decreases after the age of 65.	298 (79.1)	22 (5.8)	57 (15.1)
The risk of osteoporosis is higher in men than in women.	296 (78.5)	49 (13.0)	32 (8.5)
Thin women are at a lower risk of osteoporosis.	218 (57.8)	76 (20.2)	83 (22.0)
Ethnicity affects the risk of osteoporosis.	195 (51.7)	94 (24.9)	88 (23.4)
A high body mass index increases the risk of osteoporosis.	76 (20.2)	150 (39.8)	151 (40.0)
**Knowledge regarding nutrition**			
Calcium-rich foods are effective for preventing osteoporosis.	377 (100.0)	0 (0.0)	0 (0.0)
Exposure to sun prevents osteoporosis.	314 (83.3)	31 (8.2)	32 (8.5)
Taking Vitamin C using food sources is very effective for the prevention of osteoporosis.	227 (60.2)	54 (14.3)	96 (25.5)
Animal protein intake is not related to osteoporosis.	188 (49.9)	75 (19.9)	114 (30.2)
**Knowledge regarding physical activity**			
Lacking enough physical activity increases the risk of osteoporosis.	312 (82.8)	31 (8.2)	34 (9.0)
Walking helps prevention of osteoporosis.	288 (76.4)	41 (10.9)	48 (12.7)
Activities like running and jumping, which exert much force on the feet, help improve bone health.	209 (55.4)	109 (28.9)	59 (15.7)

#### Associations Between Knowledge and Sociodemographic Characteristics

There was no association between scores of knowledge and any sociodemographic characteristic (*p* > 0.05).

### Preventive Practices

#### Dietary Habits

The median (IQR) intakes of fruits [2 (1–2) vs. 2–4], and meats and eggs [1.7 (1.4–2.2) vs. 1–2], in servings/day were adequate compared to the recommendations of the Iranian food pyramid (Table 4). In contrast, those of dairy products [1.5 (0.9–2.3) vs. 2–3], nuts and legumes [0.5 (0.2–1.1) vs. 1], and vegetables [0.3 (0.1–1) vs. 3–5] were inadequate compared to the recommendations. In addition, the median (IQR) intake of tea and/or coffee was 1 (0.6–2), and that of cola was 0.1 (0–0.3), in servings/day, which were considered limited.

**TABLE 4:** Intakes of the food groups/items and frequencies of consumption of the food items among the teachers.

Food group/item	Median (IQR) intake (servings/day)	Frequency of consumption, n (%)			
		Daily	Weekly	Monthly	Never
**Dairy products**	1.5 (0.9–2.3)				
Milk, yogurt, and/or dough	1.0 (0.3–1.0)	180 (47.9)	154 (41.0)	28 (7.4)	14 (3.7)
Cheese	0.7 (0.2–0.7)	187 (49.7)	160 (42.6)	25 (6.6)	4 (1.1)
Ice cream	0.1 (0.0–0.2)	25 (6.7)	185 (49.2)	119 (31.6)	47 (12.5)
**Nuts and legumes**	0.5 (0.2–1.1)				
Nuts	0.1 (0.1–0.3)	88 (23.4)	173 (46.0)	98 (26.1)	17 (4.5)
Legumes	0.2 (0.1–0.5)	71 (18.9)	275 (73.1)	29 (7.7)	1 (0.3)
**Fruits**	2.0 (1.0–2.0)	276 (73.4)	85 (22.6)	15 (4.0)	0 (0.0)
**Vegetables**	0.3 (0.1–1.0)	105 (27.9)	208 (55.3)	54 (14.4)	9 (2.4)
**Meats and egg**	1.7 (1.4–2.2)				
Chicken	0.6 (0.6–0.9)	32 (8.5)	321 (85.4)	17 (4.5)	6 (1.6)
Red meat	0.3 (0.3–0.6)	5 (1.3)	267 (71.0)	86 (22.9)	18 (4.8)
Seafood	0.1 (0.1–0.3)	4 (1.1)	248 (65.9)	100 (26.6)	24 (6.4)
Egg	0.3 (0.1–0.5)	98 (26.1)	256 (68.1)	20 (5.3)	2 (0.5)
**Others**					
Tea and/or coffee	1.0 (0.6–2.0)	263 (70.0)	62 (16.5)	23 (6.1)	28 (7.4)
Cola	0.1 (0.0–0.3)	28 (7.4)	135 (35.9)	106 (28.2)	107 (28.5)

#### Associations Between Dietary Habits and Sociodemographic Characteristics

The median intakes of dairy products, fruits, and nuts were significantly higher among female teachers than among males: 1.5 servings/day vs. 1.2, *p* < 0.05; 2 vs. 1, *p* < 0.05, and 0.14 vs. 0.05, *p* < 0.05; respectively. Conversely, the median intakes of chicken, egg, and cola were significantly higher among male teachers than among females: 0.9 servings/day vs. 0.6, *p* < 0.05; 0.4 vs. 0.2, *p* < 0.001, and 0.3 vs. 0.1, *p* = 0.001; respectively. There was no association between intakes of any food items/groups and age, years’ experience, marital status, academic degree, the field of study, or university (*p* > 0.05).

#### Associations Between Knowledge and Dietary Habits

There was no association between the score of knowledge regarding nutrition or responses to its knowledge items and intakes of the related food groups/items (*p* > 0.05).

#### Physical Activity

The physical activity level of 121 (32.1%) teachers was high, 124 (32.9%) moderate, and 135 (35%) low. Participation of the teachers was the highest in moderate house and yard chores, and somehow in walking for transportation among all types of physical activity (Table 5).

**TABLE 5:** Physical activity values of the teachers and participation of them in different types of physical activity.

Type of physical activity	Median (IQR) minutes/week	Median (IQR) METs-minute/week	Participation, n (%)
**Total walking**	30 (0–180)	99 (0–594)	
Transportation	10 (0–95)	33 (0–314)	168 (55.4)
Leisure-time	0 (0–40)	0 (0–132)	127 (33.7)
Working-time	0 (0–0)	0 (0–0)	16 (4.2)
**Total moderate physical activity**	280 (85–800)	960 (305–2900)	
Moderate house chores	120 (35–360)	360 (105–1080)	323 (85.7)
Moderate yard chores	50 (0–180)	200 (0–720)	260 (69.0)
Vigorous yard chores	0 (0–20)	0 (0–110)	113 (30.0)
Leisure-time	0 (0–0)	0 (0–0)	48 (12.7)
Working-time	0 (0–0)	0 (0–0)	19 (5.0)
Cycling	0 (0–0)	0 (0–0)	7 (1.9)
**Total vigorous physical activity**	0 (0–0)	0 (0–0)	
Leisure-time	0 (0–0)	0 (0–0)	49 (13.0)
Working-time	0 (0–0)	0 (0–0)	17 (4.5)
**Total physical activity**	420 (130–1005)	1614 (462–3780)	

#### Associations Between Physical Activity and Sociodemographic Characteristics

The percentage of the high physical activity level was significantly higher among the teachers who studied physical education or humanities and social sciences than others: 68.8 and 41%, respectively, vs. 29.4% and lower; *p* < 0.01. The median METs-minute/week of leisure-time vigorous and total vigorous physical activities were significantly higher among teachers who had studied physical education than among those who had studied education, humanities and social sciences, formal sciences, or natural sciences: 86.4 and 320, respectively, vs. 0; *p* < 0.05. The median METs-minute/week of moderate house chores was significantly higher among female teachers than among males: 360 vs. 120, *p* < 0.001. The median METs-minute/week of walking for transportation was significantly higher among teachers who were never-married (297) than among those who were married (33) or separated (0) (*p* < 0.01 and *p* < 0.05, respectively). There was no association between physical activity levels or METs-minutes/week and age, years’ experience, academic degree, or university (*p* > 0.05).

#### Associations Between Knowledge and Physical Activity

There was no association between the score of knowledge regarding the physical activity or responses to its knowledge items and values of any types of physical activity (*p* > 0.05), except in two cases. First, the mean METs-minute/week of leisure-time vigorous physical activity was significantly higher among teachers who knew about the role of enough physical activity in the prevention of osteoporosis than among others: 180.2 vs. 48, *p* < 0.05. Second, the mean METs-minute/week of leisure-time walking was significantly higher among teachers who knew about the role of weight-bearing activities in the prevention of osteoporosis than among others: 157.5 vs. 95.1, *p* < 0.05.

## Discussion

Knowledge of the teachers about osteoporosis was moderate, with a mean overall knowledge score of 13.3 out of 19. In previous studies, low and moderate knowledge with mean scores of 18.4 out of 30 and 18 out of 24 were reported among female teachers of Rafsanjan and Shahr-e-Kord, respectively (20, 21). All of the teachers knew about the role of calcium-rich foods, and the majority knew about the role of sun exposure, enough physical activity, and walking in the prevention of osteoporosis. Similarly, the majority of the female teachers of Rafsanjan and Shahr-e-Kord knew about the role of sun exposure and regular physical activity (20, 21). However, only about half of the teachers in this study knew about the role of animal protein intake and weight-bearing activities in prevention. On the one hand, this indicates that sources of knowledge about osteoporosis, to which the teachers have been nearly exposed, have extensively focused on the role of the first group of items in preventing osteoporosis. On the other hand, this may indicate that these sources do not usually discuss the second group of items, although these items also have essential roles in the prevention. Therefore, the sources are not fully informative, are still old, and need to be updated.

Preventive practices of the teachers about osteoporosis were somewhat adequate concerning dietary habits. Among the food groups that improve bone health, intakes of fruits, meats, and eggs were adequate, while those of dairy products, nuts and legumes, and vegetables were inadequate. Among the food items that may have adverse effects on bone health, intakes of tea and/or coffee and cola were limited. In some previous studies conducted among different populations, low intake was reported for dairy products (31–33), vegetables (32), coffee, and cola (31, 34). However, in some others, moderate intake was reported for green leafy vegetables (31), and high intake was reported for tea (34). These variations in the results could be either due to the differences between populations or questionnaires used to recall dietary habits in the studies.

The preventive practices of the teachers were moderate concerning physical activity, with a median total physical activity of 420 min in a week. In contrast, in previous studies, low physical activity was reported among Iranian female heads of household, working women of Karachi, and the over-40 population of Malaysia. These studies reported mean physical activity of 210.9 min/week, 85.6% of inactive or minimally active participants, and 90.7% of participants with no regular program for exercise, respectively (10, 32, 33). These differences in the populations and the method or categorization of physical activity assessment could explain the diversity in the results.

Despite the high knowledge regarding nutrition and the somewhat acceptable practices concerning dietary habits, there was no association between these two variables for dairy products, fruits, vegetables, and meats and eggs in this study. Similarly, in the study conducted among women of Karachi, in spite of having knowledge, the participants were not practicing appropriate lifestyle and dietary habits (32). Three reasons may explain the results of the present study. First, the teachers may not know the food sources rich in the macro or micronutrients that improve bone health. Second, they may not know the recommended amounts for these food sources. Third, like many other groups, the teachers may have a common dietary habit, which is not associated with knowledge about osteoporosis regarding nutrition. Rather, it may be associated with some of their sociodemographic characteristics, the habits developed during their life, or the time they can spend on programming a healthy dietary habit. In line with this point, intakes of dairy products, fruits, nuts, chicken, egg, and cola were associated with sex in this study. Similarly, in the study conducted among the over-40 Malaysian population, dairy and coffee/tea intakes were associated with sex (33). Also, in line with the point, seafood intake seems to be higher among the citizens of the coastal cities like Bandar-Abbas than those of other cities. The mean intake of seafood, in servings/week, was higher among elementary school teachers of Bandar-Abbas in this study (1.6) than among 30–50-year-old women of Arak, Iran in a previous study (35).

Despite the moderate level of both knowledge and practices regarding physical activity, there was no association between these two variables for almost all types of physical activity, except leisure-time vigorous and walking physical activities in this study. In a previous study, no significant relationship was found between knowledge and practice of weight-bearing activities toward osteoporosis among the Kuala Lumpur population (31). However, in another study, over-40 Lebanese women with a self-reported regular physical activity lasting less than 10 min compared to those exercising between 10 and 20 min had reached lower knowledge scores (36). Two groups of explanations may provide the reason for the results of the present study. First, moderate house and yard chores and walking for transportation, with the highest participation of the teachers among all types of physical activity, are generally inseparable parts of daily life. Therefore, they may only be associated with some sociodemographic characteristics rather than knowledge about their role in preventing osteoporosis. In line with this explanation, the METs-minute/week of moderate house chores and walking for transportation were associated with sex and marital status, respectively, in this study. Also, the physical activity status of the over-40 Malaysian population was associated with sex and age (33). On the other hand, working-time and vigorous physical activities are inseparable parts of the daily life of physical education teachers and may be associated with this field of study rather than knowledge about their role in preventing osteoporosis. In line with this explanation, the METs-minute/week of these physical activities were significantly higher among the physical education teachers than among other teachers in this study. Similarly, the physical activity status of the over-40 Malaysian population was significantly different based on the nature of their job (33).

The second group of explanations is about the association of knowledge regarding physical activity with METs-minute/week of leisure-time vigorous and walking physical activities. These types of physical activity, like many other leisure-time activities, need motivation, time, and support to get done. One motivating factor can be knowledge about the role of these physical activities in the prevention of diseases such as osteoporosis.

### Strengths and Limitations

This study had some strengths compared to some previous studies. First, this study assessed the dietary habits of the teachers for a wide variety of food items improving bone health. Second, in this study, nothing was asked about taking calcium or vitamin D supplements, neither in the items of knowledge nor in the items of practice. Asking about these supplements may convey the wrong message that all individuals need to take these supplements to improve their bone health and prevent osteoporosis (11).

The limitation of this study was that the questionnaire was online and self-administered, and thus, it is possible that some reported information is not accurate. This could be either due to any problems with filling in the online form of the questionnaire or due to any misunderstanding of the questionnaire’s items.

## Conclusion

Knowledge of the elementary school teachers about osteoporosis was moderate, and their preventive practices were somewhat adequate concerning dietary habits and moderate concerning physical activity. This study suggests that the teachers’ preventive practices may be mainly associated with their sociodemographic characteristics, the habits developed during their life, or the time they can spend on programming a healthy lifestyle. Therefore, the health system needs to motivate and support the teachers for improving their practices, and high knowledge, *per se*, does not help accomplish this goal.

## Data Availability Statement

The raw data supporting the conclusions of this article will be made available by the authors, without undue reservation.

## Ethics Statement

The studies involving human participants were reviewed and approved by the Ethical Committee of Shahid Beheshti University of Medical Sciences, Tehran, Iran. The patients/participants provided their written informed consent to participate in this study.

## Author Contributions

A-AK and AN contributed to the conceptualization and data collection of the study, and reviewed and edited the draft. AN performed the statistical analyses and wrote the first draft of the manuscript. A-AK provided resources and supervision. Both authors contributed to the article and approved the submitted version.

## Conflict of Interest

The authors declare that the research was conducted in the absence of any commercial or financial relationships that could be construed as a potential conflict of interest.

## Publisher’s Note

All claims expressed in this article are solely those of the authors and do not necessarily represent those of their affiliated organizations, or those of the publisher, the editors and the reviewers. Any product that may be evaluated in this article, or claim that may be made by its manufacturer, is not guaranteed or endorsed by the publisher.
