# Supplementary material for: Knowledge and Preventive Practices About Osteoporosis Among Elementary School Teachers of Bandar-Abbas in 2020
Source: Front Nutr. 2022 Apr 4;9:849639. doi: 10.3389/fnut.2022.849639 (PMC9014264; doi:10.3389/fnut.2022.849639)
Supplement: Supplementary file 1 [file Presentation_1.PDF]

Assessment of Knowledge and Preventive Practices about Osteoporosis among School Teachers

Dear teacher,

This questionnaire is developed to assess your knowledge and preventive practices about osteoporosis. It takes you around 15 minutes to fill in this questionnaire. Thank you in advance for your cooperation in helping us prioritize the educational programs about this disease.

Sociodemographic characteristics

1- Gender: 1. Man □ 2. Woman □	2- Age: …… years	3- Height: …… cm
4- Weight: …… kg	5- Academic degree:	6- Field of study:
7- University:	8- Years' experience: …… years	
9- Marital status: 1. Never-married □ 2. Engaged □ 3. Married □ 4- Separated □ 5. Widowed □ 6. Other (state it)		

**Knowledge items**	**True**	**False**	**Do not know**
1- The risk of osteoporosis is higher in men than in women.	□	□	□
2- Ethnicity affects the risk of osteoporosis.	□	□	□
3- Smoking increases the risk of osteoporosis.	□	□	□
4- Exposure to the sun prevents osteoporosis.	□	□	□
5- Calcium-rich foods are effective for preventing osteoporosis.	□	□	□
6- Taking Vitamin C using food sources is very effective for preventing osteoporosis.	□	□	□
7- Thin women are at a lower risk of osteoporosis.	□	□	□
8- Lacking enough physical activity increases the risk of osteoporosis.	□	□	□
9- Osteoporosis is also named "Rheumatism."	□	□	□
10- A blood test must be used for the diagnosis of osteoporosis.	□	□	□
11- The chance of developing osteoporosis is higher in women with early menopause.	□	□	□
12- The risk of osteoporosis decreases after the age of 65.	□	□	□
13- Osteoporosis increases the risk of bone fracture.	□	□	□
14- Osteoporosis does not usually have a clear sign until the first bone fracture occurs.	□	□	□
15- A high body mass index increases the risk of osteoporosis.	□	□	□
16- Walking helps prevention of osteoporosis.	□	□	□
17- Animal protein intake is not related to osteoporosis.	□	□	□
18- Activities like running and jumping, which exert much force on the feet, help improve bone health.	□	□	□
19- In a person with osteoporosis, a previous bone fracture increases the chance of another bone fracture.	□	□	□

Dietary habits

Food items and their units of measurement are listed in the first column. For each food item, if you consume it daily, write the daily intake per unit of measurement in the column "Daily." If you do not consume the food item daily, write the weekly intake per unit of measurement in the column "Weekly." If you do not consume the food item daily or weekly, write the monthly intake per unit of measurement in the column "Monthly." If you never consume the food item, write '0' in the column "Never."

**Food items (unit of measurement)**	**Daily**	**Weekly**	**Monthly**	**Never**
1. Milk, yogurt, and/or dough (glass)				
2. Cheese (matchbox size)				
3. Ice cream (glass)				
4. Nuts – almonds, walnuts, etc. (table spoon)				
5. Legumes – beans, peas, etc. (table spoon)				
6. Fruits (medium apple)				
7. Vegetables (glass)				
8. Chicken (medium chicken thigh)				
9. Red meat (how many times?)				
10. Seafood (hand palm size)				
11. Egg (how many?)				
12. Drinks like tea and coffee (glass)				
13. Cola (glass)				

International Physical Activity Questionnaire

This questionnaire will ask you about the time you spent being physically active in the last 7 days. Vigorous physical activities refer to activities that take hard physical effort and make you breathe much harder than normal. Moderate activities refer to activities that take moderate physical effort and make you breathe somewhat harder than normal.

Section 1: The first section is about your physical activity at work. In this regard, answer the question below.

1. Are you a physical education teacher, or do you have a second job needing physical activity?
Yes □ (the job title:) No □ (Skip to Section 2, after question 7)

Pay attention that questions below do not include traveling to and from work.

2. During the last 7 days, on how many days did you do vigorous physical activities like heavy lifting or climbing upstairs as part of your work? (Think about only those physical activities that you did for at least 10 minutes at a time.) …… days per week No vigorous job-related physical activity □ (Skip to question 4)

3. How much time did you usually spend on one of these days doing vigorous physical activities as part of your work? …… hours per day …… minutes per day

4. Again, think about only those physical activities that you did for at least 10 minutes at a time. During the last 7 days, on how many days did you do moderate physical activities like carrying light loads as part of your work? (Please do not include walking.)<br>…… days per week No moderate job-related physical activity □ (Skip to question 6)
5. How much time did you usually spend on one of these days doing moderate physical activities as part of your work? …… hours per day …… minutes per day
6. During the last 7 days, on how many days did you walk for at least 10 minutes at a time as part of your work? Please do not count any walking you did to travel to or from work.<br>…… days per week No job-related walking □ (Skip to Section 2)
7. How much time did you usually spend on one of these days walking as part of your work?<br>…… hours per day …… minutes per day
**Section 2:** These questions are about how you traveled from place to place, including to places like work, stores, movies, and so on.
8. During the last 7 days, on how many days did you travel in a motor vehicle like a train, bus, car, or tram?<br>…… days per week No traveling in a motor vehicle □ (Skip to question 10)
9. How much time did you usually spend on one of these days traveling in a train, bus, car, tram, or other kind of motor vehicle? …… hours per day …… minutes per day
10. During the last 7 days, on how many days did you bicycle for at least 10 minutes at a time to go from place to place? …… days per week No bicycling from place to place □ (Skip to question 12)
11. How much time did you spend on one of these days to bicycle from place to place?<br>…… hours per day …… minutes per day
12. During the last 7 days, on how many days did you walk for at least 10 minutes at a time to go from place to place? …… days per week No walking from place to place □ (Skip to Section 3)
13. How much time did you spend on one of these days walking from place to place?<br>…… hours per day …… minutes per day
**Section 3:** This section is about some of the physical activities you might have done in the last 7 days in and around your home, like housework, gardening, yard work, general maintenance work, and caring for your family.
14. Think about only those physical activities that you did for at least 10 minutes at a time. During the last 7 days, on how many days did you do vigorous physical activities like heavy lifting in the garden or yard?<br>…… days per week No vigorous activity in garden or yard □ (Skip to question 16)
15. How much time did you usually spend on one of these days doing vigorous physical activities in the garden or yard? …… hours per day …… minutes per day
16. Again, think about only those physical activities that you did for at least 10 minutes at a time. During the last 7 days, on how many days did you do moderate activities like carrying light loads, sweeping, washing car, and raking in the garden or yard?<br>…… days per week No moderate activity in garden or yard □ (Skip to question 18)

17. How much time did you usually spend on one of these days doing moderate physical activities in the garden or yard? …… hours per day …… minutes per day
18. Once again, think about only those physical activities that you did for at least 10 minutes at a time. During the last 7 days, on how many days did you do moderate activities like carrying light loads, washing windows, scrubbing floors and sweeping inside your home?<br>…… days per week No moderate activity inside home □ (Skip to Section 4)
19. How much time did you usually spend on one of these days doing moderate physical activities inside your home? …… hours per day …… minutes per day
**Section 4:** This section is about all the physical activities that you did in the last 7 days solely for recreation, sport, exercise or leisure. Please do not include any activities you have already mentioned.
20. Not counting any walking you have already mentioned, during the last 7 days, on how many days did you walk for at least 10 minutes at a time in your leisure time?<br>…… days per week No walking in leisure time □ (Skip to question 22)
21. How much time did you usually spend on one of these days walking in your leisure time?<br>…… hours per day …… minutes per day
22. Think about only those physical activities that you did for at least 10 minutes at a time. During the last 7 days, on how many days did you do vigorous physical activities like aerobics, jump-roping, running, fast bicycling, fast swimming, or soccer in your leisure time?<br>…… days per week No vigorous activity in leisure time □ (Skip to question 24)
23. How much time did you spend on one of these days doing vigorous physical activities?<br>…… hours per day …… minutes per day
24. Again, think about only those physical activities that you did for at least 10 minutes at a time. During the last 7 days, on how many days did you do moderate physical activities like bicycling at a regular pace, swimming at a regular pace, walking fast, and volleyball in your leisure time?<br>…… days per week No moderate activity in leisure time □ (Skip to Section 5)
25. How much time did you spend on one of these days doing moderate physical activities?<br>…… hours per day …… minutes per day
**Section 5:** These questions are about the time you spend sitting while at work, at home, while doing course work and during leisure time. This may include time spent sitting at a desk, visiting friends and relatives, reading or sitting or lying down to watch television. Do not include any time spent sitting in a motor vehicle that you have already told me about.
26. During the last 7 days, how much time did you usually spend sitting on a weekday?<br>…… hours per day …… minutes per day
27. During the last 7 days, how much time did you usually spend sitting on a weekend day?<br>…… hours per day …… minutes per day

Thank you for your help!
